# Encapsulation of Alcohol Dehydrogenase in Mannitol by Spray Drying

**DOI:** 10.3390/pharmaceutics6010185

**Published:** 2014-03-24

**Authors:** Hirokazu Shiga, Hiromi Joreau, Tze Loon Neoh, Takeshi Furuta, Hidefumi Yoshii

**Affiliations:** 1 Department of Applied Biological Science, Kagawa University, 2393 Ikenobe, Miki, Kita, Kagawa 761-0795, Japan; E-Mails: shiga@ag.kagawa-u.ac.jp (H.S.); tlneoh@gmail.com (T.L.N.); 2 Department of Biotechnology, Graduate School of Engineering, Tottori University, 4-101 Koyama Minami, Tottori 680-8552, Japan; E-Mail: furuta@sun.ocn.ne.jp; 3 Department of Food Engineering, ENSBANA University of Burgundy, 1 Esplanade Erasme-21000 Dijon, France; E-Mail: foodeng.kagawa@gmail.com

**Keywords:** alcohol dehydrogenase, encapsulation, spray drying, mannitol

## Abstract

The retention of the enzyme activity of alcohol dehydrogenase (ADH) has been studied in various drying processes such as spray drying. The aim of this study is to encapsulate ADH in mannitol, either with or without additive in order to limit the thermal denaturation of the enzyme during the drying process. The retention of ADH activity was investigated at different drying temperatures. When mannitol was used, the encapsulated ADH was found inactive in all the dried powders. This is presumably due to the quick crystallization of mannitol during spray drying that resulted in the impairment of enzyme protection ability in comparison to its amorphous form. Maltodextin (dextrose equivalent = 11) was used to reduce the crystallization of mannitol. The addition of maltodextrin increased ADH activity and drastically changed the powder X-ray diffractogram of the spray-dried powders.

## 1. Introduction

In pharmaceutical formulation researches, the drying process is often used in order to stabilize and preserve biologically active molecules such as enzymes contained in solutions. Among the conventional drying processes that rely on evaporation, spray drying is a well-established method for processing liquids into powders. Unlike freeze drying, spray drying utilizes heat from a hot gas stream to evaporate microdispersed droplets created by atomization of a continuous liquid feed, offering faster, less harsh, and cost-effective solvent evaporation, hence it is more adapted to produce enzyme powder [1]. Nevertheless, heat is required to evaporate water, causes irreversible chemical structural changes due to thermal denaturation [2,3,4]. Broadhead *et al.* [5] studied the effect of drying temperature on the activity loss of β-galactosidase during spray drying. They found that higher outlet temperature of the drying air resulted in extensive enzyme denaturation. Therefore, it is important to control the drying temperature in order to minimize the activity loss of enzymes.

Mannitol, a nonreducing sugar alcohol of mannose and an isomer of sorbitol, is widely used for tablet formulation in the pharmaceutical industry and food products [6]. Alcohol sugars can interact with the surface of proteins, and stabilize them thermodynamically against heat- and cold-denaturation [7]. To explain this stabilization two hypotheses are advanced, either by molecular interactions, such as hydrogen bonds [8], or by trapping the enzyme in a glassy matrix [9]. But in these two cases, at least a part of sugar alcohol has to co-exist with protein in a single amorphous phase in order to stabilize the protein. However, it is also well known that mannitol has a strong tendency to crystallize. The vial breakage phenomenon is a striking illustration of this tendency [10]. Many studies tried to maintain mannitol under its amorphous state by avoiding the crystallization. Several cases were tested by adding proteins, salts, sugars or acids [11].

To obtain a spray-dried powder, it is necessary to evaporate the water by a thermal process. However, high inlet air temperatures often cause irreversible denaturation of enzymes contained in feed solutions during spray drying. Alcohol dehydrogenase (ADH) encapsulation by spray drying was investigated using mannitol as well as a mixture of mannitol and maltodextrin (MD) as the wall matrix. The retention of ADH activity was investigated at different drying air temperatures. The effect of MD on the retention of ADH activity was also investigated. The morphology of the spray-dried particles was also observed with a scanning electron microscope (SEM).

## 2. Materials and Methods

### 2.1. Chemicals

The basic excipient of all feed solutions was mannitol. Mannitol (C_6_H_14_O_6_, *M*_r_ = 182.17) was purchased from Mitsubishi Shoji Foodtech Company (Tokyo, Japan). ADH, NAD^+^ dependent form yeast, and NAD^+^ were obtained from Oriental Yeast Company (Osaka, Japan). The additive was maltodextrin (MD, Pindex #2, dextrose equivalent = 11) purchased from Matsutani Chemical Industry (Hyogo, Japan).

### 2.2. Preparation of Feed Enzyme Solutions

Feed solutions of 400 g were prepared using either 10 wt% pure mannitol or 20 wt% of the equal-mass mixture of mannitol and MD (mixture of 10 wt% of mannitol and 10 wt% of MD) in order to encapsulate 200 mg of ADH.

ADH was also dissolved in distilled water, agitated for a few minutes before being added to the previously obtained mannitol solution. The feed solution obtained at this point was slowly mixed at least for another 30 min. Once fully mixed, the final feed solution was spray dried immediately.

### 2.3. Spray-Drying of Enzyme Solutions

A mono-pump (type CY04F, Heishin Ltd., Kobe, Japan) was used to feed the enzyme solution to an Ohkawara L-8 spray dryer (Ohkawara Kakouki, Yokohama, Japan) equipped with a rotary disc atomizer. The spray dryer was composed of a cylindrical chamber with an 800 mm internal diameter and 564 mm height, followed by a conical chamber of 564 mm high with a 60° angle. The powder was separated off the stream of hot air in a cyclone and collected directly into a metallic pot.

Operational conditions of the spray dryer were set as follows: rotation speed of atomizer: 20,000 rpm; feed flow rate: 20 mL/min; air flow rate: 130 kg/h; inlet air temperature varies over a range of 60–130 °C; outlet air temperature, varies with inlet temperature.

The powder samples collected after spray drying were kept in a glass bottle tightly capped and placed in plastic boxes with silica gel to impede moisture absorption. They were then stored in a refrigerator to minimize thermal damages to the enzyme until use.

### 2.4. Water Content in Powder

The water content in the powders was determined by thermal gravimetric analysis (TGA) instrument (TG/DTA 6200, Exstar 6000, SII, Tokyo, Japan). About 10 mg of spray-dried powder was put in an aluminum pan which was heated up to 300 °C at a rate of 10 °C min^−1^. Duplicate measurement was performed.

### 2.5. Assay of Alcohol Dehydrogenase (ADH) Activity and Retention of ADH

To measure the activity of ADH before and after spray drying, a spectrophotometry method (spectrophotometer model V-560, Jasco, Tokyo, Japan) was used based on the following reaction:

Ethanol + NAD^+^ → Acetaldehyde + NADH + H^+^

The samples were prepared by dissolving 40 mg of spray-powder in 960 mg of water. The sample of pure ADH solution was made with 5 mL of water and 5 mg of ADH. The pure ADH solution was diluted ten times to obtain a concentration of 0.1 mg/mL of ADH which is equivalent to the concentration of ADH in the solution of the prepared formulation. To minimize the degradation of ADH during measurement, the samples were put on an ice-containing insulated box until measurement. Five microliters of these solutions were diluted with 1 mL of 10.9 mM sodium pyrophosphate pH 8.8 reaction buffer containing 10 mM NAD^+^ and 1.13 M ethanol. The absorption time course of NADH at 340 nm was recorded for 1 min with a computer using Spectra Manager software (JASCO Corporation, Hachioji, Japan) that gave directly the activity (U/mL) of ADH. The extinction coefficient of NADH used was 6.31 × 10^3^ L mol^−1^ cm^−1^. The retention of ADH activity was calculated by dividing the activity of ADH in spray-dried powder by the activity of ADH in the feed solution.

Spray drying was carried out once or twice for each operational condition (inlet temperature and MD addition). For every sample of spray-dried powder, two samples were collected and the ADH activity was measured in triplicate or quadruplicate for every sample.

### 2.6. Powder Particle Size Analysis

Laser diffraction particle size analyzer (SALD 2000-model, Shimadzu, Kyoto, Japan) was used to measure the particle size distribution of spray-dried powder. One tenth of a gram of the powder was mixed with 2 mL of buffer (dioctyl sodium sulfosuccinate, 10 mmol/L) and agitated for 1 min with a vortex mixer and then 15 min with a sonicator. Then, a few microliters of this mixture was put into the SALD 2000 cell to measure the particle size.

### 2.7. Morphological Characterization of Powders by Scanning Electron Microscopy (SEM)

The external structures of spray-dried particles were observed by SEM (JSM-6060, JEOL Datum Ltd., Tokyo, Japan). The powder was placed on the SEM stubs using double sided adhesive tape (Nisshin EM Co. Ltd., Tokyo, Japan). The specimens were subsequently coated with Pt–Pd using a magnetron sputter coater (MSP-1S, Vacuum Device, Mito, Japan). The coated samples were then analyzed using the SEM operating at an accelerating voltage of 2.0 kV.

### 2.8. X-ray Diffractogram

The X-ray diffractogram patterns of the spray-dried powders were measured with a Rigaku X-ray diffractometer (RINT-TTR III, Tokyo, Japan). The powder was densely packed in an aluminum sample holder. The operation conditions of the diffractometer were as follows: X-ray target, Cu; filter, Ni CuKa-radiation; voltage, 30 kV; time constant, 1 s; scanning speed, 2 °/min; divergence slit, 1 mm; receiving slit, 0.5 mm; count full scale, 16,000 c/s.

## 3. Results and Discussion

### 3.1. Effect of Outlet Air Temperature on the Retention of ADH Activity and Water Content in Spray-Dried Powders

**Figure 1:**
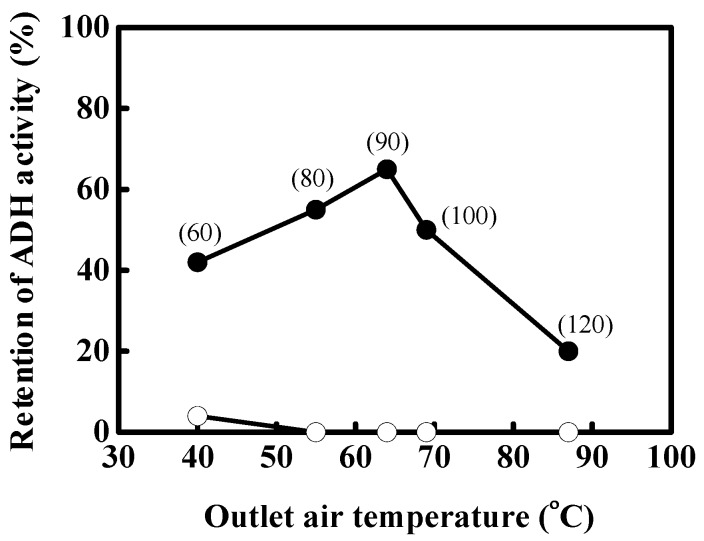
Effect of outlet air temperature on the retention of alcohol dehydrogenase (ADH) activity in spray-dried powders with mannitol (○) and the equal-mass mixture of mannitol and maltodextrin (MD) (●). The value in the parenthesis is the inlet air temperature.

The retention of ADH activity was investigated at inlet air temperature of 60, 80, 90, 100 and 120 °C. The corresponding average outlet temperatures to the inlet air temperature were 40, 55, 63, 69, and 87 °C, respectively. Figure 1 shows the effect of outlet air temperature on the retention of ADH activity in spray-dried powders with mannitol and the equal-mass mixture of mannitol and MD. Under these conditions of spray drying, the spray-dried mannitol with ADH (open symbol) showed no enzymatic retention. No activity was detected in the spray-dried powders, possibly as a result of low water content in spray-dried mannitol and the rapid crystallization of mannitol. Mannitol is used for spray drying as it permits the preparation of a powder at low inlet temperatures (60 °C). However, it lacks the ability to stabilize ADH.

The addition of MD to mannitol increased the retention of ADH activity (closed symbol). This might be due to the inhibition of mannitol crystallization by MD and the increase of solid content. As shown in Figure 1, the retention of ADH activity was significantly influenced by the drying air temperature because the enzyme was subjected to crystal stress and thermal stress [2,3,4]. The increment of outlet air temperature might have affected the crystal formation rate with the increase in mannitol solubility. This result revealed an optimal inlet air temperature around 90 °C with a maximal retention of 66%. After this optimal temperature, the retention decreased quickly to 20% because of the irreversible denaturation of ADH when droplets are subjected to high drying air temperatures [5].

**Figure 2:**
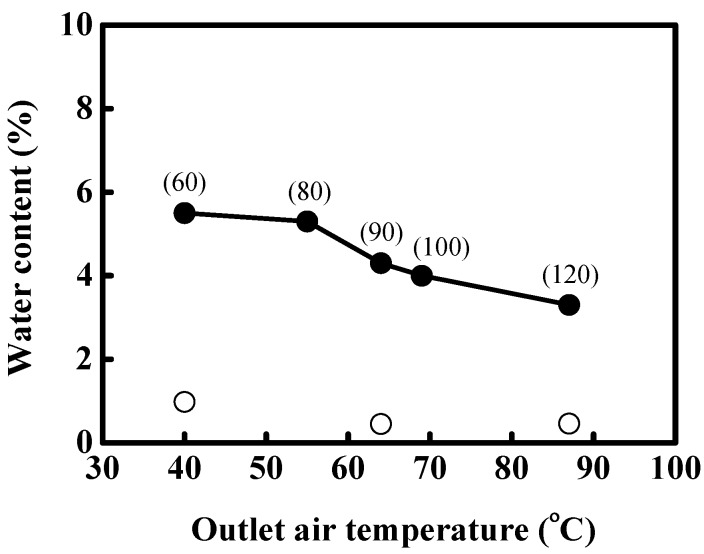
Effect of outlet air temperature on the water content in spray-dried powders with mannitol (○) and the equal-mass mixture of mannitol and MD (●).

Figure 2 shows the effect of outlet air temperature on the water content of the spray-dried powders with mannitol and the equal-mass mixture of mannitol and MD. One of the main properties of a spray-dried powder is its water content, which is affected by the various spray drying conditions. Generally, lower water content in the powder can be obtained at higher outlet air temperature. For all spray-dried powders of mannitol, an interestingly low water content was measured (<1%). This may be due to the low hygroscopic property of mannitol [12]. However, the water content of spray-dried powders with the equal-mass mixture of mannitol and MD increased by the addition of MD for the various outlet temperatures, and decreased with an increase in outlet air temperature. This result indicates that the mixed excipient of mannitol with other ingredients is a useful wall matrix for preparation of spray-dried powder at high outlet air temperatures of above 60 °C, which is generally the temperature for the increase of protein denaturation.

### 3.2. Effect of Maltodextrin (MD) on the Crystal Structure and Morphology of Spray-Dried Powder

**Figure 3:**
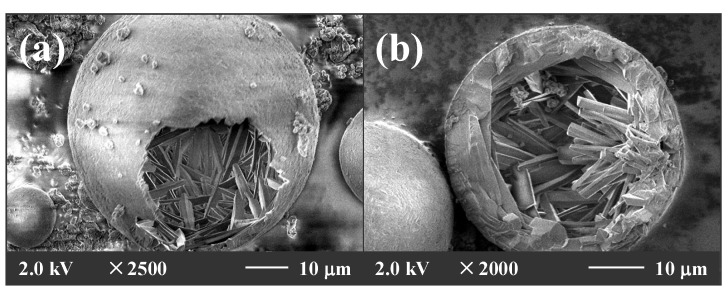
Crystallized particles of mannitol at the outlet air temperature at (**a**) 40 °C and (**b**) 66 °C.

Figure 3 shows the formation of particles containing needle-shaped crystals of mannitol observed by SEM.

**Figure 4:**
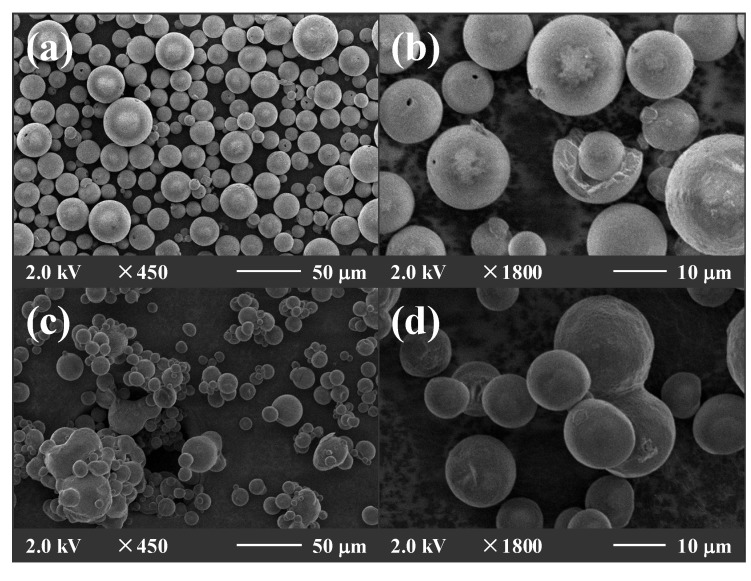
External structures of spray-dried powders. Inlet air temperature, 120 °C; atomizer speed, 20,000 rpm; (**a**,**b**), mannitol; (**c**,**d**), the equal-mass mixture of mannitol and MD. (**a**) and (**c**) are 450 times magnification; (**b**) and (**d**) are 1000 times magnification.

The particles containing crystallized mannitol were observed by SEM as shown in Figure 3. The formation of needle-shaped crystal was observed in the mannitol spray-dried at the outlet air temperatures of 40 and 66 °C. Figure 4 shows the external structures of spray-dried powders with mannitol and the equal-mass mixture of mannitol and MD at the inlet air temperature of 120 °C and an atomizer speed of 20,000 rpm. The SEM microphotographs illustrate that the particles agglomerated with the addition of MD due to the higher hygroscopicity of the equal-mass mixture of mannitol and MD. The surface of the particles was smooth and no shriveled particles could be found in all powders. In the use of mannitol as a wall matrix, low hygroscopicity of mannitol and high water diffusion coefficient in mannitol solution might facilitate water diffusion from the spray droplets and prevent the nucleation of air bubble. Therefore, no vacuoles could form in the spray-dried powders. 

**Figure 5:**
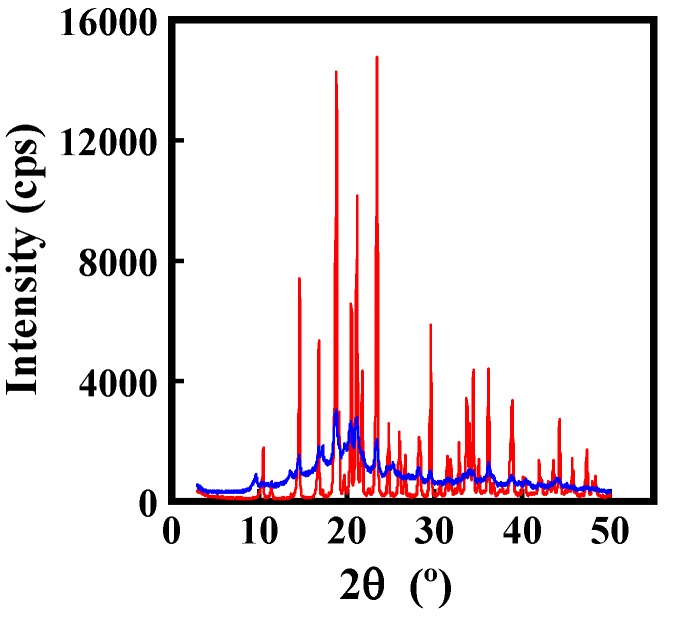
Powder X-ray diffractograms of spray-dried powders. Red line: mannitol; and blue line: the equal-mass mixture of mannitol and MD.

The mannitol spray-dried powder was found to crystallize with well-defined peaks as shown by the X-ray diffraction patterns in Figure 5. The intensities of the X-ray peaks decreased significantly for the sample that contained equal-mass mixture of mannitol and MD. These crystal structures might have affected the retention of ADH activity.

**Figure 6:**
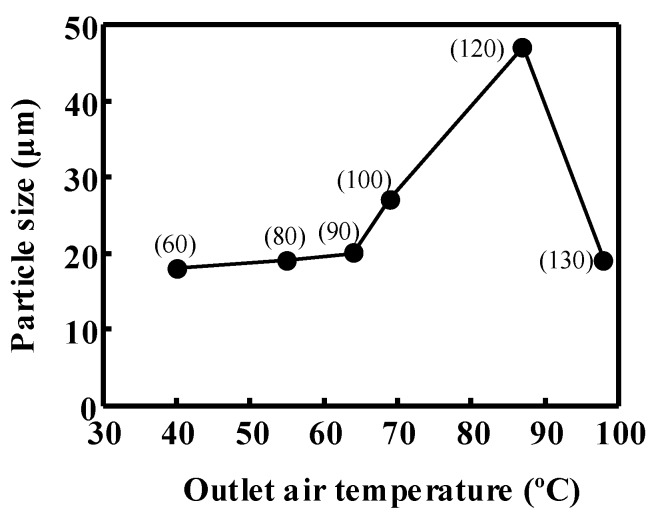
Effect of outlet air temperature on the powder size. The wall matrix is the equal-mass mixture of mannitol and MD.

The particle size of the spray-dried mannitol powder was about 20 µm for all the outlet air temperatures studied. However, particle sizes of spray-dried powder containing the equal-mass mixture of mannitol and MD were bigger when the outlet temperatures were between 66 and 90 °C as shown in Figure 6. The powder size increased until about 50 µm.

### 3.3. Effect of Outlet Air Temperature on Powder Recovery

**Figure 7:**
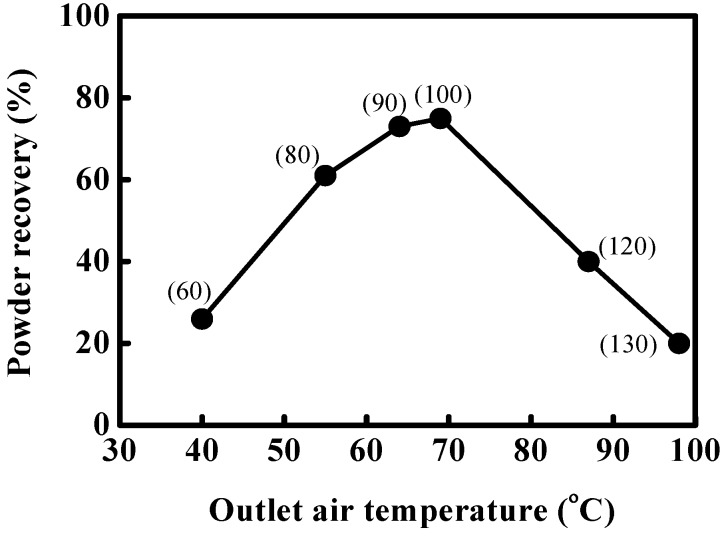
Effect of outlet air temperature on the powder recovery for formulation with equal-mass mixture of mannitol and MD.

With mannitol as the wall matrix, the powders stuck on the inside walls of the spray dryer and pipes. Only few grams of spray-dried powder were recovered in the sample pot of the spray dryer, taking the average recovery percentage down to 3.7%. The rest was recovered by scraping the inside walls of the spray dryer and pipes with a brush, but still, only an average of 47% of the material could be recovered. Figure 7 represents the recovery of material (in %) as a function of the outlet air temperature for the equal-mass mixture of mannitol and MD. The corresponding inlet air temperature is shown in parentheses. The recovery of material is maximal (nearly 75%) for the corresponding outlet air temperature of 70 °C. Adhikari *et al.* [12] measured a similar recovery of material in the spray drying of sucrose with MD used as a drying aid. The powder was very adhesive and so the recoveries of powder were lower. In use of sucrose as wall matrix at glass transition temperature, 67 °C, the recovery of spray-dried powder was remarkably low since the wall matrix became very adhesive and aggregated. The powders with the equal-mass mixture of mannitol and MD as wall material aggregated at outlet temperatures above 70 °C. Powder morphology and enzyme activity were affected by the temperature difference between outlet temperature and glass transition temperature of the equal-mass mixture of mannitol and MD. It is interesting to notice the increase of powder recovery from the outlet temperature of 40 to 64 °C (the optimal outlet temperature obtained with the inlet temperature of 90 °C). At inlet temperatures above 90 °C, the particle structure might have collapsed, resulting in agglomeration of powder and crystals of mannitol. The outlet temperature is an important operation factor for obtaining high enzyme activity and powder recovery in spray-dried powders with mannitol as the wall matrix.

## 4. Conclusions

ADH was encapsulated in mannitol with or without MD. Although mannitol allows production of ADH powders at low temperatures (60 °C), rapid crystallization of mannitol also poses problems to the retention of ADH activity. When only mannitol was used, the encapsulated ADH was found to be inactive in all the spray-dried powders. With the addition of MD to mannitol, the ADH activity of the spray-dried powder was improved. At the optimum outlet air temperature of 64 °C, the retention of 66% of ADH activity was achieved. The outlet air temperature also affects the powder recovery.
